# Clinical Profile of First-Time Diagnosis of Autism Spectrum Disorder in School-Aged Youth

**DOI:** 10.3390/children13091220

**Published:** 2026-09-09

**Authors:** Kimberly Burkhart, Alyssa Palumbo, Kristen Sanford, Anna Olczyk, Nori Minich

**Affiliations:** 1 University Hospitals, Cleveland, OH 44106, USA; kristen.sanford@uhhospitals.org (K.S.); anna.olczyk@uhhospitals.org (A.O.); 2 School of Medicine, The Case Western Reserve University, Cleveland, OH 44106, USA; nori.minich@uhhospitals.org; 3 College of Medicine, The Ohio State University, Columbus, OH 43210, USA; alyssa.palumbo@osumc.edu

**Keywords:** autism spectrum disorder, first-time diagnosis, school-aged, sex differences

## Abstract

**Highlights:**

**What are the main findings?**

- ASD Level 1 in school-aged children presents a distinct clinical profile marked by high rates of co-occurring conditions (ADHD and anxiety), a discrepancy between cognitive and adaptive behavioral functioning, and informant-dependent symptom severity—all of which may contribute to diagnostic overshadowing and delay, especially in females.
- Caregivers consistently rated internalizing symptoms and externalizing behaviors higher than teachers, which may be explained, in part, by the majority of children currently receiving academic accommodations/modifications, possibly minimizing symptoms in the school setting.
- Both caregivers and teachers rated social communication/motivation deficits as more impairing in females than males.

**What are the implications of the main findings?**

- Referrals were primarily physician-driven, with approximately one-fourth receiving current medication management. Receiving routine symptom management may inadvertently contribute to diagnostic delay.
- Effective identification and support for school-aged children with ASD Level 1 requires looking beyond core autism symptoms alone toward a broader, multi-informant, multidomain clinical picture for earlier identification and to ensure that the full scope of co-occurring needs are met.

**Abstract:**

Background/Objectives: There is limited research on clinical profiles of school-aged children receiving a first-time diagnosis of autism spectrum disorder (ASD) Level 1 and mixed findings related to sex differences in phenotype. This study aimed to describe the clinical profile of school-aged children receiving a first-time diagnosis of ASD Level 1, explore sex differences in phenotype by symptom severity and symptom domain profiles, and compare parent and teacher ratings on standardized measures of social, emotional, and behavioral functioning. Methods: A retrospective chart review of an ASD assessment clinic was completed. Eighty-one school-aged children were diagnosed with ASD Level 1. Measures of social, emotional, adaptive, and behavioral functioning were completed. Results: Females were significantly older at the time of diagnosis (M = 9.7 years) in comparison to males (M = 8.5 years). Additionally, over half of children diagnosed with ASD Level 1 presented with ADHD and over a third presented with an anxiety disorder. Approximately one-fourth of those diagnosed were currently taking psychotropic medication, and a substantial proportion had reported speech or language delay. Approximately half presented with food selectivity and sleep problems, with only 38% currently receiving behavioral health therapy services. Caregivers reported significantly higher internalizing symptoms and externalizing behavior on all Achenbach scales in comparison to teachers. Caregivers also reported significantly greater autism-specific social concerns. No statistically significant sex differences were found in parent or teacher ratings of domain scores. Based on caregiver reports, males demonstrated greater severity of aggressive behavior. Females displayed greater deficits in social communication and motivation based on both parent and teacher reports. Overall cognitive ability fell in the average range, while adaptive behavioral functioning was in the moderately low range. Conclusions: ASD Level 1 in school-aged children presents a distinct clinical profile marked by high rates of co-occurring conditions (ADHD and anxiety), existing academic accommodations/modifications, physician referral for evaluation, and varied symptom severity by informant type, all of which may contribute to diagnostic overshadowing and delay, especially in females.

## 1. Introduction

Autism spectrum disorder (ASD) is defined by persistent deficits in social communication and interaction, along with restrictive or repetitive patterns of behavior. Symptomology must present early in development and significantly impair daily functioning [1,2]. ASD prevalence has increased notably over the past two decades among U.S. children. According to the Centers for Disease Control and Prevention (CDC) surveillance data, prevalence rates rose from approximately 1 in 150 in 2000 to approximately 1 in 31 in 2025 [3].

ASD is largely polythetic in nature, resulting in a range of symptom severity characterized by differing levels of support. Youth with more severe phenotypic presentations tend to be evaluated and diagnosed in early childhood due to prominent deficits in social communication and interaction and restricted interests and repetitive behaviors that significantly impact day-to-day functioning, requiring very substantial support. Pediatric populations with milder forms of ASD, categorized as Level 1, are less likely to be identified early [3]. This population is colloquially defined as those with high-functioning autism (HFA). HFA is not a term found in the Diagnostic and Statistical Manual of Mental Disorders-5 (DSM-5) but is sometimes utilized by clinicians to describe individuals with ASD that present without intellectual impairment (IQ > 70) [4]. In the U.S., as of 2022, about 40% of children aged 8 years with ASD were diagnosed with an intellectual disability [3,4], indicating that the majority of those diagnosed with ASD are characterized as having Level 1 impairment.

There is a paucity of research on the clinical profiles of school-aged children receiving a first-time diagnosis of ASD Level 1, as much of the existing literature has focused on early childhood [5]. Recent longitudinal work demonstrates that ASD diagnostic status in children with an IQ above 70 may change between early childhood and school age, highlighting school age as a clinically meaningful period for the identification and characterization of ASD [6]. Children without intellectual disability are diagnosed significantly later than those with, often after they have entered grade school, as they may not appear to meet the standard diagnostic criteria in early childhood. In a 2020 report from the Autism and Developmental Disabilities Monitoring Network (ADDM), children without an intellectual disability were diagnosed on average 10–12 months later than those with an intellectual disability [7]. In subsequent ADDM surveillance cycles, the median age at the first documented ASD diagnosis has been approximately 47 months, with reported ranges spanning roughly 36 to 70 months [3]. This delay may be partly due to the low sensitivity of standard screening tools for ASD Level 1, causing delays in diagnosis [8].

Additionally, the concept of “coupling,” when children with ASD present with co-occurring conditions, obscures a clear differential diagnosis and can lead to diagnostic overshadowing, with many coupled conditions sharing similar ASD-related traits [9,10]. For example, anxiety disorder can often overshadow an ASD diagnosis as both may present with impairments in social skills [9]. Additionally, ASD can be overlooked as obsessive-compulsive disorder (OCD) as restricted interests can be confused with compulsions [9]. Furthermore, attention deficit hyperactivity disorder (ADHD), a common co-morbidity among children with ASD, is often diagnosed first and may serve as a misleading explanation for behaviors that are better attributed to autism [11,12,13,14].

A recent study examining autism phenotypes and genetic correlates in children aged 4 to 18 found four phenotype classes: social and behavioral challenges, mixed ASD with developmental delay, moderate challenges, and broadly affected [15]. The social and behavioral challenges group (approximately 37% of the study sample) typically met developmental milestones at a similar timeframe to those without ASD, but exhibited social challenges, restricted interests and repetitive behaviors (RRBs), as well as higher rates of comorbid ADHD, OCD, anxiety, and depression. This group has been linked to genetic mutations that appear later in childhood, which may explain the pattern of limited developmental delays and later autism diagnosis. Other phenotype groups such as the broadly affected group (approximately 10% of the study sample) were characterized by more extreme challenges in socialization, communication, RRBs and exhibited developmental delays. This group was linked to a high number of de novo mutations, which are genetic mutations that are not inherited by their parents but appear for the first time in the individual. Due to the severe nature and early onset of their symptoms, children in the broadly affected group were more likely to receive an autism diagnosis earlier in childhood. The mixed ASD with developmental delay group, on the other hand (approximately 19% of study sample), was characterized by developmental delays and fewer comorbidities with anxiety, depression, and disruptive behaviors and had a greater number of rare inherited genetic variants [15]. This study highlights the heterogeneity in ASD presentation and genetic contribution, which can make accurate and timely diagnosis of ASD difficult.

Diagnostic delays are more common in females than males, with ASD reported to disproportionately impact males [16]. Possible reasons for this delay include environmental and sociocultural factors that shape the developing brain, biological factors such as possible in utero hormonal influences, and/or maternal immune activation [17,18,19,20]. Neuroimaging studies have attempted to define ASD sex differences in volumetric characteristics and connectivity patterns; all yielding conflicting results [21,22,23]. Regardless of the lack of consensus surrounding differences in the origination of female and male etiology, it is important to note that current diagnostic and screening tools have been developed based on the male phenotypic presentation [17,21,24,25,26,27]. Recent work also suggests that cognitive ability moderates sex differences in ASD, with females without intellectual disability displaying fewer observable autistic features [28].

The lack of knowledge regarding the female phenotype, particularly among individuals with ASD Level 1, has contributed to missed or delayed diagnosis and under-treatment. Earlier studies reported that caregivers requested evaluations for females with ASD several years later than for males, with females often evaluated at school age (approximately 8 years) compared to males evaluated earlier in childhood (approximately 5 years) [29,30,31,32]. These findings align with contemporary ADDM surveillance data, which show wide variability in age at ASD diagnosis and continued identification of many children later than in early childhood [3].

Studies that have attempted to define the female phenotype report that females present with more awareness of social codes of conduct as they may more easily develop coping strategies to help mimic conventional behaviors, or camouflage, compared to males [16]. However, other studies report that females are more impaired in terms of social interaction and communication skills than their male counterparts [33]. These inconsistencies may again be due to many standardized autism measures validated largely on male samples, which may underestimate autistic traits in females due to differences in restricted interests, masking behaviors, and symptom expression [34,35]. Additional reported observations include that HFA males exhibit avoidant eye contact, whereas HFA females display hyper-fixated eye contact. HFA females are also noted to have sensory symptoms, lower inflexibility to adherence to routines or ritualized patterns, and fixated interests that appear similar to typically developing peers [16,27]. A 2025 systematic review and meta-analysis by Suominen et al. [36] found that, overall, adolescent females with ASD showed significantly higher rates of anxiety than males with ASD, with differences becoming more pronounced as age and IQ increase. These findings suggest that school-aged children with ASD Level 1 may have a distinct clinical profile, particularly as adolescence brings heightened sensitivity to peer influence, increased exposure to rejection, and transitions into more demanding environments (e.g., moving from elementary to middle school), all of which can catalyze the development of internalizing symptoms.

With regards to ADHD, males with ASD are more likely to be diagnosed with co-occurring ADHD than females [37,38,39,40]. This disproportionate impact of ADHD among males with ASD has been attributed, in part, to sex-related differences in symptom presentation. Males’ ADHD symptoms may be more overt due to greater externalization, often presenting as hyperactivity and impulsivity, whereas females’ ADHD symptoms are more likely to be internalized and characterized by inattentiveness, which may reduce the likelihood of referral for evaluation [38,39,41]. However, there is no clear consensus regarding these presentation differences, and the underlying etiologies of sex-related differences in ADHD diagnosis among individuals with ASD remain unclear [41]. Importantly, Suominen et al. [36] emphasize that inconsistencies in the broader literature on sex differences may largely stem from substantial methodological heterogeneity across studies. Variability in diagnostic criteria, sampling strategies, age range, measurement tools, operational definitions of anxiety and other disorders, and the degree to which masking behaviors are considered all contribute to divergent findings. In addition to the aforementioned challenges, small sample sizes and the frequent reliance on clinical rather than community-based samples make it challenging to draw clear, comparable conclusions about sex differences in ASD.

In addition to clinician evaluation, parent and teacher data add important insights into a child’s behavior and functioning. The current literature shows that parent and teacher reports rarely coincide with differences pronounced in ASD populations due to contextual variability of symptom expression [42,43,44]. Some reports suggest that this is due to parents identifying greater social and behavioral issues [45], while others report teachers identifying greater problems [44,46]. Additional parent and teacher discrepancies exist when looking at ASD sex differences, with reports of parents denoting greater concerns for females compared to males, and teachers recording greater concerns for males compared to females. The differences between parent and teacher reports appear even greater the older the child [47,48]. It is worth noting that these discrepancies are amplified for females with ASD, as their enhanced ability to camouflage in order to mimic their typically developing peers is more commonly observed in the school setting, where many of a child’s social interactions take place [49].

Given the limited research on clinical profiles of school-aged children receiving a first-time ASD Level 1 diagnosis, as well as mixed findings on sex differences in phenotype, this study aims to: (1) describe the clinical profile of this population; (2) explore sex differences in phenotype based on symptom domains and severity; and (3) compare parent and teacher ratings on standardized measures of social, emotional, and behavioral functioning, using the Achenbach system and the Social Responsiveness Scale, Second Edition. Exploratory analyses will examine whether children taking psychotropic medications differ on these measures and if outcomes vary by insurance type. This study contributes to the literature by focusing specifically on school-aged youth, including near-equal representation of public and commercial insurance, physician referral as the primary entry point, and the inclusion of both parent and teacher ratings along with data on cognitive and adaptive behavioral functioning.

## 2. Materials and Methods

A retrospective chart review spanning 3 years (September 2022–September 2025) was completed for children who presented to a school-aged (5–13 years) ASD assessment clinic at a Midwest academic medical center. The authors’ institutional review board deemed this research exempt. A total of 145 children were assessed in the clinic. Of those, 81 were diagnosed with ASD Level 1 requiring support, with the majority being male (*N* = 58; 72%) and having commercial insurance (*N* = 44; 54%). Sex was defined based on medical record documentation (male/female). Although gender is a distinct sociocultural construct, gender identity data were not available in this dataset; therefore, the analysis focused on sex differences. Some 12% of those assessed met criteria for ASD Level 2 and were not included in this study. To be assessed in this clinic, ASD must have been the primary presenting concern, and the patient must have been attending school. Exclusion criteria consisted of prenatal alcohol or drug exposure and/or specific learning disorder as primary presenting concerns. Please see Table 1 for demographic information.

### 2.1. Procedures

Caregivers contacted a developmental-behavioral pediatrics and psychology clinic at a Midwestern academic medical center to have their child assessed for ASD. Caregivers completed and returned an intake packet indicating presenting concerns, involvement in therapy services, and any significant birth or medical history. If the patient was determined to meet inclusion criteria, the intake specialist scheduled the child for an evaluation. The evaluation was completed by both a developmental-behavioral pediatrician and a psychologist (total assessed, *N* = 42; ASD Level 1 diagnosis, *N* = 20) or psychologist independently. Developmental-behavioral pediatric and psychology fellows assisted with evaluation. Evaluation took place over the course of two testing sessions, with the third session scheduled as a parent-only feedback session. All patients were diagnosed using the same instruments/measures and DSM-5 criteria. All examiners were clinically trained in the administration of the Autism Diagnostic Observation Scheduled, Second Edition (ADOS-2). A parent-only diagnostic intake interview was completed to assess the presence of ASD DSM-5 diagnostic criteria. Achenbach scales (Child Behavior Checklist and Teacher Report Form), Vanderbilt forms (caregiver and teacher), Social Responsiveness Scale, Second Edition (caregiver and teacher) and the Vineland Adaptive Behavior Scale, Third Edition (caregiver), were completed. The Kaufman Brief Intelligence Test, Second Edition (KBIT-2), and ADOS-2 were also administered.

### 2.2. Measures

Achenbach Scales (Child Behavior Checklist (CBCL) and Teacher’s Report Form (TRF): The CBCL and TRF [50] are standardized global emotional and behavioral rating forms used for children aged 1.5–5 years and 6–18 years that compare parent and teacher reports of a child’s behavior with those of other children of the same sex and age. The CBCL and TRF each contain 113 items, which assess emotional and behavioral problems over the previous 6 months, with three response options (0 = not true, 1 = somewhat or sometimes true, 2 = very true or often true). The questionnaires yield eight syndrome scales: Anxious/Depressed, Withdrawn/Depressed, Somatic Complaints, Social Problems, Thought Problems, Attention Problems, Rule-Breaking Behavior, and Aggressive Behavior; two higher order factors: Internalizing and Externalizing; a Total Score; and six DSM-oriented scales consistent with DSM diagnostic categories: Depressive Problems, Anxiety Problems, Somatic Problems, Attention Deficit/Hyperactivity Problems, Oppositional Defiant Problems, and Conduct Problems for children aged 6–18. The CBCL and TRF use T-scores with a mean of 50 and a standard deviation of 10. T-scores between 65 and 69 indicate borderline clinical elevation, whereas T-scores of 70 or higher indicate clinical elevation. The CBCL and TRF have been found to demonstrate good validity and reliability [50]. Deckers et al. [51] also found some evidence to support the use of the CBCL and TRF as screeners for ASD.

Vanderbilt ADHD Rating Scales (VARS): The VARS includes parent- and teacher report forms based on the Diagnostic and Statistical Manual of Mental Disorders, Fourth Edition Text Revision (DSM-IV, TR) [52]. The Vanderbilt ADHD Diagnostic Parent Rating Scale (VADPRS) is a 55-item parent-report measure and the Vanderbilt ADHD Diagnostic Teacher Rating Scale (VADTRS) is a 43-item teacher report measure, which assesses symptoms of ADHD and common comorbid conditions in children aged 5–12 years [53,54]. The majority of the items are rated on a 4-point scale ranging from “0” never to “3” very often with the performance items rated on a 5-point scale ranging from “1” excellent to “5” problematic. Psychometric properties for the VADPRS and VADTRS indicate acceptable validity and reliability [53,54].

Social Responsiveness Scale, Second Edition (SRS-2): The SRS-2 [55] is a 65-item rating scale used to identify social impairment associated with autism spectrum disorder and quantify its severity in children aged 4–18 (school-aged form). The school-aged form can be used by parents and teachers. Items are scored on a 4-point Likert scale ranging from “1” not true to “4” always true. Results are reported as T-scores, with a mean of 50 and a standard deviation of 10. A T-score of 59 and below is considered to be within normal limits. A T-score between 60 and 65 is considered in the mild range (deficiencies in reciprocal social behavior that are clinically significant and may lead to mild to moderate interference with everyday life) while a T-score between 66 and 75 is considered to be in the moderate range (deficiencies in reciprocal social behavior that are clinically significant and lead to substantial interference with everyday social interactions; such scores are typical for children with autism spectrum disorders of moderate severity). A T-score of 76 or higher is considered to be in the severe range and is associated with a clinical diagnosis of autism spectrum disorder. Items cluster into six subdomains that correspond to an overarching two-factor structure of DSM-IV-TR diagnostic domains: Social Communication and Interaction (Social Awareness, Social Cognition, Social Communication, and Social Motivation subdomains) and Restrictive Interests and Repetitive Patterns, as well as a total score. The SRS-2 is considered a reliable and well-validated tool [56].

The Vineland Adaptive Behavior Scales, Third Edition (Vineland-3), Caregiver Rating Form, Domain-Level: The Vineland-3 [57] assesses the functioning of individuals from birth to age 90 in their day-to-day activities across four domains and provides a composite score that summarizes the individual’s performance across all domains. In the domain-level form, there are 180 items in a Likert-type format, with scores ranging from 0 (never) to 2 (usually or often) to indicate the frequency with which the individual performs a certain behavior without help or prompting when needed. Some items also require a response of yes (scored as 2) or no (scored as 0). Scores that fall in the 85–115 range are considered average/typical. Scores falling above 116 are either moderately high or high. Scores falling between 70 and 84 are considered moderately low or delayed, and scores falling 69 and below are considered low or significantly delayed. The Vineland-3 demonstrates acceptable validity and reliability [58].

Kaufmann Brief Intelligence Test, Second Edition, Revised (KBIT-2): The KBIT-2 [59] is a brief assessment of verbal and nonverbal intelligence in individuals aged 4–90 years and comprises three subtests (Verbal Knowledge, Matrices, Riddles). The KBIT-2 produces three scores: Verbal IQ (VIQ), Nonverbal IQ (NVIQ), and an IQ composite score, which are reported as standard scores (M = 100, SD = 15). Research supports its validity and reliability as an estimate of cognitive ability [60].

Autism Diagnostic Observation Schedule, Second Edition (ADOS-2): The ADOS-2 [61] is a semi-structured, standardized assessment of communication, social interaction, and play or imaginative use of materials for individuals who have been referred for possible autism or autism spectrum disorder. The ADOS-2 consists of five modules, which are selected based on the examinee’s expressive language level and age. Observations noted in the assessment inform specific coded items for each module pertaining to (a) language and communication, (b) reciprocal social interaction, (c) play and imagination, (d) stereotyped behaviors and restricted interests, and (e) other behaviors. Items in these domains are scored on a scale of 0–3, with higher scores indicating greater symptom severity related to autism. The ADOS-2 demonstrates high reliability [62].

## 3. Results

### 3.1. Statistical Analysis

We used descriptive statistics to summarize patient characteristics, paired-samples *t*-tests to compare parent and teacher data, and independent *t*-tests to compare sex differences and explore possible differences in report form scores based on medication use and insurer type. For measures that were not normally distributed, significant differences were confirmed with non-parametric tests.

### 3.2. Patient Characteristics

There was a significant difference in age at the time of diagnosis, with females (9.7 years) being older than males (8.5 years), *t* (79) = −2.260, *p* = 0.03. The majority of patients self-identified as White (79%) and were referred by their primary care clinician (44.4%) or self-referred (27.2%). Nearly one-fourth (23.5%) were on medication to manage psychiatric symptoms at the time of assessment. Additionally, many caregivers reported delays in developmental milestones such as delays in speech (42%), fine motor functioning (20%), and gross motor functioning (7%). Approximately 11% of those assessed reported both fine and gross motor functioning delays. Caregivers also reported several current concerns, including food selectivity (54%), difficulties with sleep (51%), and gastrointestinal problems (12%).

### 3.3. Comparisons Between Parent and Teacher Reports

Results showed significant differences in parent and teacher reports of symptoms across all Achenbach and SRS-2 subscales/scales, such that parents consistently reported higher symptom severity than the teacher report (see Table 2). On the CBCL, the mean of eight subscales was in the borderline clinically elevated range, with one scale in the clinically elevated range (i.e., Thought Problems). In contrast, based on how teachers responded to items, the mean of all subscale scores fell in the average range. On the SRS-2, based on how parents endorsed items, the mean of five of the six subscales was in the moderate range, and one subscale (Restricted Interests and Repetitive Behaviors) was in the severe range; based on how teachers endorsed items, on the other hand, the mean of four subscales was in the moderate range, with no scales in the severe range (see Table 2).

### 3.4. Comparisons Between Males and Females

There were no statistically significant sex differences found on the CBCL, TRF and parent- and teacher-reported SRS-2. There were differences between male and female ratings based on qualitative classifications (see Table 3). On the CBCL, males, on average, were rated in the borderline clinically elevated range on the Aggressive Behavior subscale, whereas females were rated in the average range. There were no differences in qualitative classifications on the TRF. On the SRS-2, parents rated females, on average, in the severe range on the Social Communication subscale and Social Communication and Interaction (SCI) scale. In contrast, males were rated in the moderate range. Teacher ratings on the SRS-2 indicated scores in the moderate range on the Social Communication and Social Motivation subscales for females, while males were rated in the mild range.

There were no sex differences based on how caregivers endorsed items on the Vineland Adaptive Behavior Scales. Overall, children scored in the moderately low range across all domains (Communication, Daily Living Skills, and Socialization). There were no sex differences on the KBIT-2 with the total IQ score, verbal composite, and nonverbal composite scores all falling in the average range (see Table 4).

Related to variables of interest on parent and teacher questionnaires, exploratory analyses revealed that caregivers whose children were on medication rated their children as having greater attention and hyperactivity/impulsivity problems, as measured by the CBCL DSM-Oriented ADHD Problems Scale, t(79) = 2.37, *p* = 0.02 (mean difference = 5.14). Teachers rated children on medication as having greater anxiety, as measured by the TRF Anxiety Scale, t(67) = 2.44, *p* = 0.02 (mean difference = 5.11) and the DSM-Oriented Depression Scale, t(67) = 2.51, *p* = 0.02 (mean difference = 4.75). In comparison to commercial insurance, children on public insurance were rated higher by their teachers on the DSM-Oriented Oppositional Defiant Problems Scale, t(66) = 2.04, *p* = 0.05 (mean difference = 4.39) and as having greater communication problems, as measured by the Vineland Adaptive Behavior Scale, t(78) = 3.13, *p* < 0.01 (mean difference = 7.79).

## 4. Discussion

The purpose of this study was to evaluate the clinical profiles of school-aged children receiving a first-time diagnosis of ASD Level 1 due to existing mixed findings related to sex differences in phenotype and in parent and teacher reports. The findings were mixed due to the sample sizes including a large age range (early childhood through adolescence) and because of the inclusion of all severity levels [3,5,32,43]. This study is novel and adds to the current literature by restricting the sample to school-aged youth receiving a first-time diagnosis of ASD Level 1, representation of public and commercial insurance, and including standardized questionnaires completed by both parents and teachers and administration of assessment measures to create a comprehensive clinical profile. Based on the present findings, ASD Level 1 females were significantly older at the time of diagnosis [16,63]. At the time of evaluation of those diagnosed with ASD Level 1, 54% presented with an existing ADHD diagnosis and 35% with an anxiety disorder diagnosis. Approximately one-fourth of those who received a first-time diagnosis of ASD Level 1 were currently taking psychotropic medication. A substantial proportion had reported speech or language delay (42%) and approximately half of those assessed currently presented with food selectivity (54%) and sleep problems (51%). It is possible that previously diagnosed common co-occurring conditions, such as ADHD and anxiety, may contribute to diagnostic delays due to symptom overlap, diagnostic shadowing, and psychotropic medication management [9,10].

In comparison to teachers, caregivers reported higher internalizing symptoms and externalizing behavior on all Achenbach scales. Nearly all CBCL syndrome scales fell in the borderline or clinically elevated range; all TRF syndrome scales, on the other hand, fell in the average range. These parent–teacher discrepancies are consistent with reports that caregivers often endorse greater emotional and behavioral concerns than teachers [45,64]. Because 59% of diagnosed children were receiving academic accommodations and/or modifications at the time of assessment, internalizing symptoms and externalizing behaviors may have been partially mitigated in the school setting, as these environmental supports could have reduced the visibility or severity of problems. It is important to note that no statistically significant sex differences emerged on symptom domain scores, likely due to these measures being norm-referenced against same-sex neurotypical peers. Relative to neurotypical samples, caregivers rated males as showing impairments in aggressive behavior and rated social communication/motivation deficits as severe in females compared with moderate in males. Teachers similarly rated social communication/motivation deficits as moderate in females and mild in males. It is possible that these differences may be explained in part by projected sex expectations rather than true differences in impairment. It is also possible that raters identify subtler but real deficits despite surface-level masking. It is also worth considering that these findings may reflect a detection bias, whereby females’ deficits reach a greater level of severity before they are recognized. Alternatively, by the time of evaluation, social communication deficits may be genuinely more pronounced in females than in males.

Overall cognitive ability fell in the average range for both sexes, while adaptive behavioral functioning was in the moderately low range. Despite residing in a highly-resourced area for mental health, only about 38% of children were receiving behavioral health therapy—possibly because nearly 25% were receiving medication management and 59% were receiving academic accommodations, which may have led caregivers and educators to perceive needs as sufficiently met. This may be especially true for children with pre-existing anxiety or ADHD diagnoses or those on psychotropic medications, potentially masking ASD-specific behaviors and reducing perceived urgency for additional services.

This study has several strengths. Much of the existing literature on ASD in the pediatric population includes ages that range from early childhood through adolescence and is not restricted to only school-aged youth, who may have their own unique phenotype. By focusing on school-aged ASD Level 1 youth, this study helps to further the understanding of this population by recognizing the factors that may contribute to delayed diagnoses and the importance of its nuances for appropriate diagnostic evaluations and supports. Furthermore, this study included females with ASD Level 1, a population that remains underrepresented in much of the existing literature. The use of parent and teacher reports is another key strength, as it provides a more comprehensive understanding of functioning across settings. Additionally, the present sample has approximately equal representation of those with commercial and public insurance. Including both groups improves generalizability among clinical populations and reduces the risk of systemic bias when interpreting findings. Both a strength and a limitation of this study is that the majority of youth were referred by a physician (pediatric primary care physician = 44.4% and psychiatrist = 16%). It is possible that diagnosis was delayed due to currently being under the care of a physician and likely receiving some type of symptom management. As a result, these findings may not generalize to other populations, such as those who are self-referred.

This study has several limitations. The sample was predominantly White (79%), limiting generalizability to more diverse populations, and included only children whose families completed the required intake paperwork, introducing potential self-selection bias. Children with possible in utero substance exposure or a primary concern of learning disorder were excluded. While the proportion of females (about one-third) is consistent with prior research [30], larger female samples are needed to further clarify sex-based differences in phenotype. Approximately one-fourth of children were taking psychotropic medication at the time of evaluation. Exploratory analyses found that children taking psychotropic medication were rated by caregivers as having clinically elevated attention and hyperactivity/impulsivity problems, and by teachers as having near borderline clinically elevated anxiety and depression. Children on public insurance were rated by teachers as showing greater oppositional defiant problems and communication problems. Future research should examine medication type and psychosocial risk factors associated with public insurance to better explain these findings, as well as item-level responses on the SRS-2 and Achenbach measures to detect potential qualitative differences in male versus female symptom expression.

## 5. Conclusions

In sum, this study demonstrates that school-aged children with ASD Level 1 may present with a clinically meaningful and distinct phenotype in terms of symptom severity and symptom domain profiles. These children exhibit high rates of co-occurring conditions, including ADHD and anxiety, along with nuanced ASD-specific behaviors that can be easily overlooked in social settings, placing them at heightened risk for diagnostic overshadowing and delayed diagnosis, particularly among females. Nearly half of the sample presented with speech or language delay, food selectivity, and sleep problems, findings that may help inform which populations to monitor closely and which additional symptoms to assess and treat. It is also notable that overall adaptive behavioral functioning fell in the moderately low range. This discrepancy between cognitive ability and day-to-day functioning across communication, socialization, and daily living skills represents an important distinction for treatment planning. Taken together, these findings underscore the need for more comprehensive assessments that look beyond autism-specific concerns, which may support earlier identification and improved access to intervention. Continued research on school-aged children with ASD Level 1 will help further define this phenotype and optimize long-term outcomes through early and appropriate intervention.

## Figures and Tables

**Table 1:** Sociodemographic characteristics of sample (*N* = 81).

	*n*	(%)
Race/Ethnicity		
White	64	(79.0)
Black	11	(13.6)
Asian	2	(2.5)
Mixed Race	4	(4.9)
Referral Source		
Self-Referral	22	(27.2)
Primary Care Physician	36	(44.4)
Psychiatrist	13	(16.0)
Therapist	7	(8.6)
Occupational Therapist	1	(1.2)
Speech Therapist	1	(1.2)
School	1	(1.2)
Pre-Existing Diagnosis		
ADHD	25	(30.9)
Anxiety	10	(12.3)
Language Disorder	3	(3.7)
Other Disorder	4	(4.9)
ADHD and Anxiety	14	(17.3)
ADHD, Anxiety, and Other	5	(6.2)
None	20	(24.7)
Academic Accommodations/Modifications	48	(59.3)
Current Service Enrollment		
Behavioral Health Services (BH)	31	(38.3)
Speech/Language Therapy	19	(23.5)
Occupational Therapy	12	(14.8)
Current Medication		
Yes	19	(23.5)
No	62	(76.5)

**Table 2:** Comparisons between parent and teacher ratings on the Achenbach scales and SRS-2.

Measure/Subscale	Parent Mean (SD)	Teacher Mean (SD)	*t*	*Df*	*p*	*d*
CBCL/TRF Syndrome Scales						
Anxious/Depressed	64.90 (10.04) *	60.01 (7.61)	3.423	68	0.001	0.412
Withdrawn/Depressed	66.32 (9.86) *	61.01 (7.50)	4.116	68	<0.001	0.496
Somatic Complaints	58.83 (8.63)	54.09 (7.08)	3.716	68	<0.001	0.447
Social Problems	67.09 (8.98) *	61.09 (8.70)	4.337	65	<0.001	0.534
Thought Problems	71.18 (6.65) **	63.73 (9.92)	5.831	65	<0.001	0.718
Attention Problems	69.28 (10.12) *	61.52 (9.49)	6.138	68	<0.001	0.739
Rule-Breaking Behavior	60.30 (7.96)	56.79 (6.68)	4.014	65	<0.001	0.494
Aggressive Behavior	65.30 (10.79) *	60.45 (9.70)	3.490	68	<0.001	0.420
CBCL/TRF DSM-Oriented Scales						
Depressive Problems	66.39 (9.37) *	58.91 (6.90)	5.797	68	<0.001	0.698
Anxiety Problems	66.50 (11.37) *	61.35 (8.48)	3.453	67	<0.001	0.419
Somatic Problems	57.83 (8.61)	54.29 (7.31)	2.677	65	0.009	0.330
Attention/Deficit	66.72 (8.67) *	61.41 (8.79)	5.075	67	<0.001	0.615
Oppositional Defiant Problems	63.50 (9.27)	59.69 (8.96)	2.841	67	0.006	0.345
Conduct Problems	61.97 (9.20)	57.56 (7.88)	4.744	65	<0.001	0.584
SRS-2						
Social Awareness	70.83 (9.35) ++	62.54 (11.71) +	4.948	58	<0.001	0.644
Social Cognition	70.66 (9.47) ++	63.93 (12.34) +	3.657	58	<0.001	0.476
Social Communication	72.16 (10.36) ++	66.09 (12.14) ++	3.167	56	0.002	0.420
Social Motivation	69.25 (11.32) ++	64.45 (11.65) +	2.553	59	0.013	0.330
Restricted Interests and Repetitive Behaviors	75.58 (8.88) +++	67.30 (13.27) ++	4.578	56	<0.001	0.606
Social Communication and Interaction	74.07 (9.32) ++	67.50 (13.44) ++	3.389	59	0.001	0.438
SRS-2 Total Score	73.93 (8.60) ++	67.43 (12.21) ++	3.627	59	<0.001	0.468

Note. ** = clinical range; * = borderline range; + = mild range; ++ = moderate range; +++ = severe range. All scaled scores were significantly different at the *p* < 0.01 level.

**Table 3:** Sex differences in parent and teacher ratings on the Achenbach scales and SRS-2.

	Parent Mean (SD)						Teacher Mean (SD)					
Measure/Subscale	Male	Female	*t*	*df*	*P*	*d*	Male	Female	*t*	*df*	*p*	*d*
CBCL/TRF Syndrome Scales												
Anxious/Depressed	65.93 (9.40) *	64.78 (11.14) *	0.470	79	0.640	0.116	59.33 (7.99)	61.70 (6.47)	−1.178	67	0.243	−0.313
Withdrawn/Depressed	67.81 (8.77) *	65.13 (11.29) *	1.019	33.243	0.315	0.280	61.08 (7.36)	60.85 (8.04)	0.115	67	0.908	0.031
Somatic Complaints	58.67 (8.44)	59.83 (8.11)	−0.562	78	0.575	−0.139	53.76 (7.08)	54.90 (7.20)	−0.607	67	0.546	−0.161
Social Problems	66.64 (8.74) *	67.43 (9.92) *	−0.353	76	0.725	−0.088	60.94 (7.94)	61.20 (10.36)	−0.113	65	0.910	−0.030
Thought Problems	71.02 (6.35) **	72.57 (7.01) **	−0.942	73	0.349	−0.236	64.43 (10.06)	62.10 (9.66)	0.877	64	0.384	0.235
Attention Problems	69.05 (9.72) *	70.48 (10.93) **	−0.575	79	0.567	−0.142	61.73 (9.90)	61.00 (8.62)	0.290	67	0.773	0.077
Rule-Breaking Behavior	61.07 (7.67)	59.30 (7.93)	0.917	75	0.362	0.228	57.39 (6.67)	55.40 (6.68)	1.115	64	0.269	0.269
Aggressive Behavior	66.79 (10.71) *	62.70 (9.66)	1.594	79	0.115	0.393	60.96 (9.44)	59.20 (10.44)	0.681	67	0.498	0.181
CBCL/TRF DSM-Oriented Scales												
Depressive Problems	67.40 (9.39) *	66.35 (9.57) *	0.451	79	0.653	0.111	58.96 (7.08)	58.80 (6.61)	0.086	67	0.932	0.023
Anxiety Problems	67.67 (10.89) *	66.04 (12.35) *	0.584	79	0.561	0.144	60.73 (9.26)	62.85 (6.19)	−1.102	52.479	0.275	−0.250
Somatic Problems	58.27 (8.48)	57.48 (7.93)	0.380	73	0.705	0.095	54.15 (7.63)	54.60 (6.67)	−0.227	64	0.821	−0.061
Attention/Deficit	66.97 (8.50) *	65.65 (8.65) *	0.624	79	0.535	0.154	61.04 (7.82)	62.30 (10.96)	−0.535	66	0.595	−0.142
Rule-Breaking Behavior	64.47 (9.48)	61.04 (8.03)	1.526	79	0.131	0.376	60.27 (8.65)	58.30 (9.76)	0.824	66	0.413	0.219
Conduct Problems	62.42 (8.75)	61.22 (10.01)	0.526	73	0.600	0.132	58.00 (7.89)	56.55 (7.98)	0.684	64	0.497	0.183
SRS-2												
Social Awareness	70.86 (8.90) ++	71.82 (11.62) ++	−0.392	76	0.696	−0.099	61.98 (11.90) +	64.53 (11.59) +	−0.779	58	0.439	−0.216
Social Cognition	69.91 (9.26) ++	72.78 (9.55) ++	−1.239	76	0.219	−0.308	63.57 (12.12) +	64.82 (13.21) +	−0.350	57	0.727	−0.101
Social Communication	71.60 (9.46) ++	76.05 (10.59) +++	−1.800	75	0.076	−0.454	65.29 (11.11) +	69.44 (14.30) ++	−1.095	26.423	0.283	−0.342
Social Motivation	68.38 (11.32) ++	71.70 (9.04) ++	−1.251	77	0.215	−0.310	63.52 (10.56) +	66.61 (13.97) ++	−0.940	58	0.351	−0.265
Restricted Interests and Repetitive Behaviors	76.24 (8.02) +++	77.05 (9.21) +++	−0.380	74	0.705	−0.096	67.33 (12.90) ++	67.24 (14.51) ++	0.023	55	0.982	0.007
Social Communication and Interaction	73.23 (8.77) ++	76.78 (9.44) +++	−1.599	77	0.114	−0.396	66.10 (12.54) ++	70.78 (15.23) ++	−1.242	58	0.219	−0.350
SRS-2 Total Score	74.20 (8.20) ++	75.86 (9.47) ++	−0.773	76	0.442	−0.195	66.48 (11.63) ++	70.58 (13.77) ++	−1.204	59	0.233	−0.333

Note. ** = clinical range; * = borderline range; + = mild range; ++ = moderate range; +++ = severe range. There are no sex differences between parent and teacher measure ratings (all *p* > 0.01).

**Table 4:** Mean scores on the Vineland-3 and KBIT-2.

	*M*	*SD*
Vineland-3		
Communication	80.54	11.71
Daily Living Skills	81.77	13.90
Socialization	73.09	12.13
Adaptive Behavior Composite	76.76	10.23
KBIT-2		
IQ	103.83	17.46
Verbal Composite	101.44	17.78
Nonverbal Composite	104.66	16.40

## Data Availability

The data presented in this study are available upon request from the corresponding author due to privacy and ethical reasons.

## Abbreviations

The following abbreviations are used in this manuscript:

ADDM	Autism and Developmental Disabilities Monitoring Network
ADHD	Attention Deficit Hyperactivity Disorder
ADOS-2	Autism Diagnostic Observation Schedule, Second Edition
ASD	Autism Spectrum Disorder
BH	Behavioral Health Services
CBCL	Child Behavior Checklist
CDC	Centers for Disease Control and Prevention
DSM-IV-TR	*Diagnostic and Statistical Manual of Mental Disorders*, Fourth Edition, Text Revision
DSM-5	*Diagnostic and Statistical Manual of Mental Disorders*, Fifth Edition
HFA	High-Functioning Autism
IQ	Intelligence Quotient
KBIT-2	Kaufman Brief Intelligence Test, Second Edition
NVIQ	Nonverbal Intelligence Quotient
OCD	Obsessive-Compulsive Disorder
RRBs	Restricted and Repetitive Behaviors
SCI	Social Communication and Interaction
SRS-2	Social Responsiveness Scale, Second Edition
TRF	Teacher Report Form
U.S.	United States
VADPRS	Vanderbilt ADHD Diagnostic Parent Rating Scale
VADTRS	Vanderbilt ADHD Diagnostic Teacher Rating Scale
VARS	Vanderbilt ADHD Rating Scales
VIQ	Verbal Intelligence Quotient
Vineland-3	Vineland Adaptive Behavior Scales, Third Edition
